# Supplementary material for: Syndecan-1 Facilitates the Human Mesenchymal Stem Cell Osteo-Adipogenic Balance
Source: Int J Mol Sci. 2020 May 29;21(11):3884. doi: 10.3390/ijms21113884 (PMC7312587; doi:10.3390/ijms21113884)
Supplement: Supplementary file 1 [file ijms-21-03884-s001.pdf]

YU *et al.* - Supplementary Figure S1

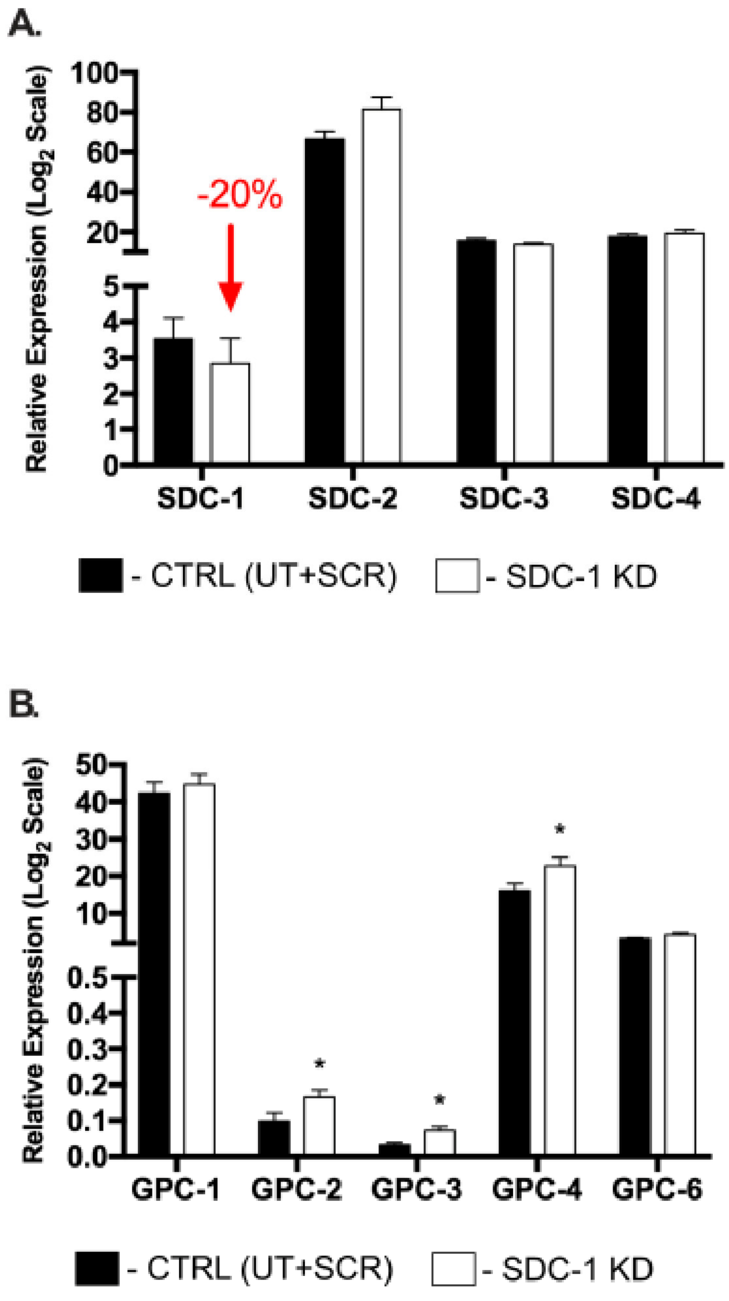

Supplementary Figure S1: Change in gene expression of heparan sulfate proteoglycan (HSPG) core proteins in undifferentiated hMSC cultures following SDC-1 knockdown (KD). Gene expression analysis was performed with RNA collected from hMSC incubated with SDC-1 specific siRNA for 72 h. **(A)** Syndecans-1-4 (SDC-1-4) core protein expression following SDC-1 KD resulted in a 20% reduction in SDC-1 expression. **(B)** Glypicans-1-4, and -6 (GPC-1-4, -6) core protein expression following SDC-1 KD. Significant differences in expression levels were detected by Student's T test, significance was set at *p<0.05. Error bars represent SEM.

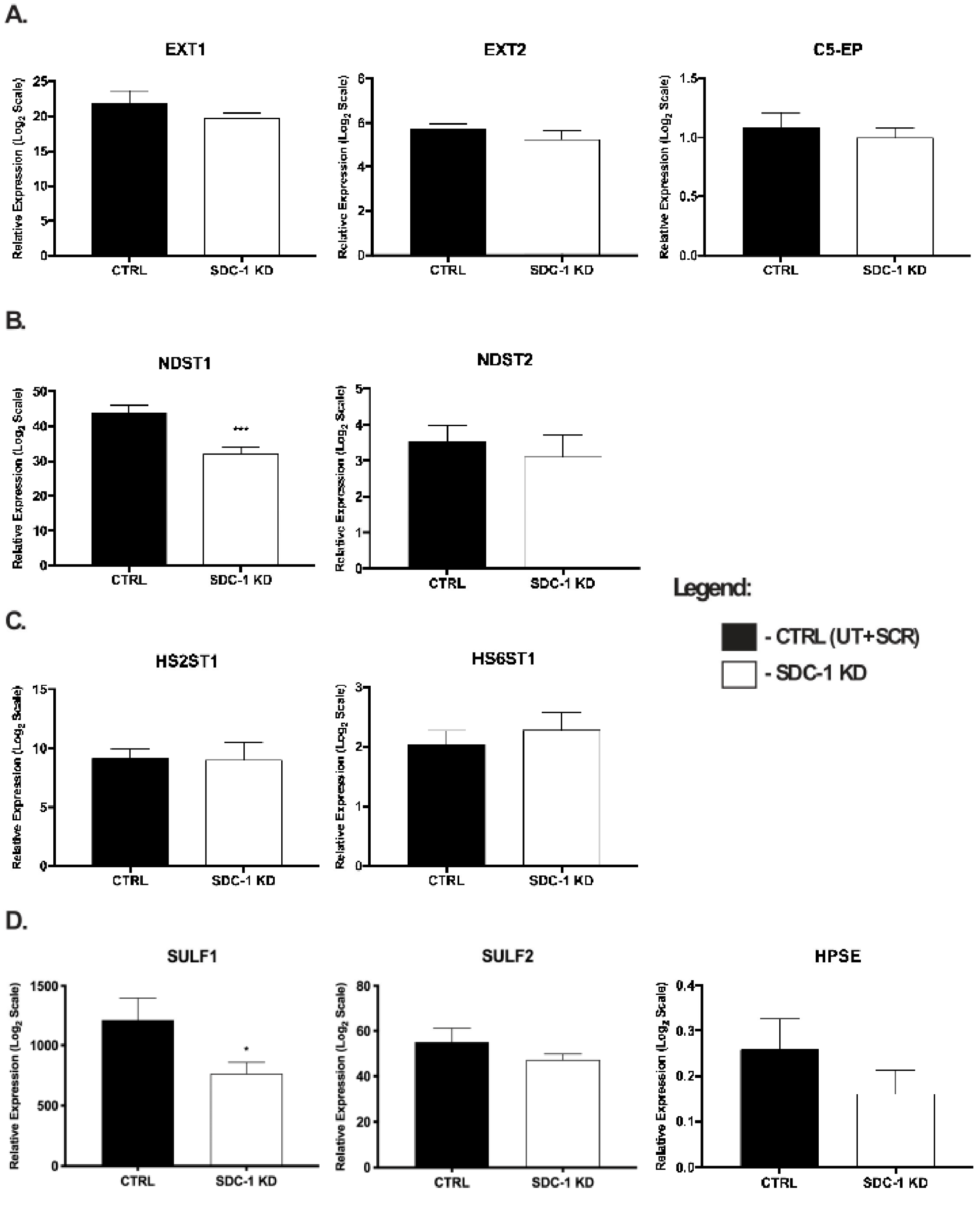

Supplementary Figure S2: Gene expression level changes in heparan sulfate biosynthesis and modification enzymes of CTRL and SDC-1 KD undifferentiated hMSC cultures. Gene expression analysis was performed with RNA collected from hMSC incubated with SDC-1 specific siRNA for 72 h. Gene expression levels of **(A)** exostosins 1-2 (EXT1-2) and C-5 epimerase (C5-EP), **(B)** *N*-deacetylase/*N*-sulfotransferases 1-2 (NDST1-2), **(C)** heparan sulfate 2-*O*-sulfotransferase 1 (HS2ST1) and heparan sulfate 6-*O*-

sulfotransferase 1 (HS6ST1), and **(D)** sulfatases 1-2 (SULF1-2) and heparanase (HPSE). Significant differences in expression levels were detected by Student's T test, significance was set at *P<0.05 and ***P<0.001. Error bars represent SEM.

YU *et al.* - Supplementary Figure S3

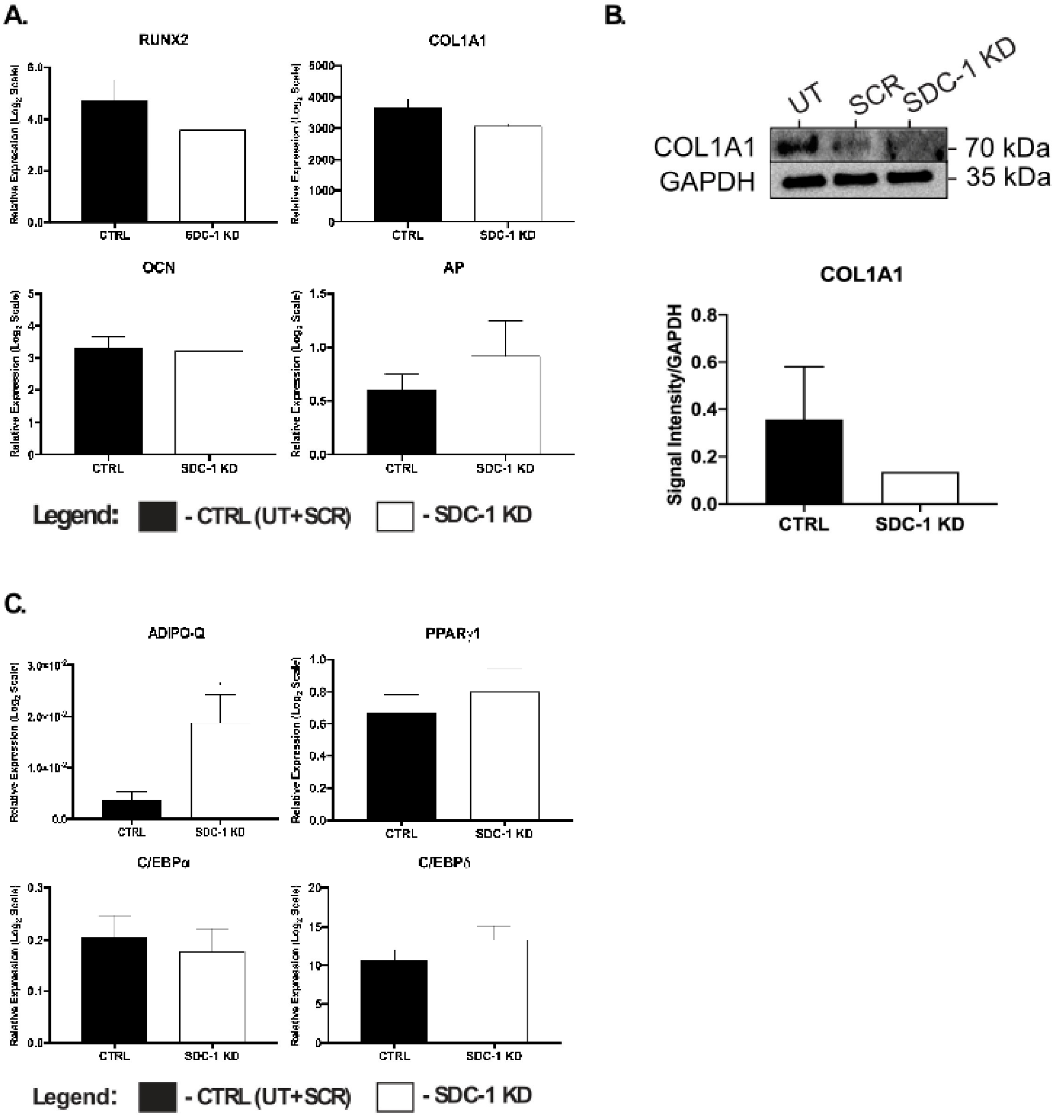

Supplementary Figure S3: Examination of osteogenic and adipogenic lineage marker expression changes in undifferentiated hMSC cultures following SDC-1 KD. Expression analysis was performed with RNA and protein collected from hMSC incubated with SDC-1 specific siRNA for 72 h and 96 h, respectively. **(A)** Gene expression changes of osteogenic markers runt-related transcription factor 2 (RUNX2), collagen, type I, alpha 1 (COL1A1), osteocalcin (OCN) and alkaline phosphatase (AP). **(B)** Western analysis of the osteogenic marker COL1A1 in undifferentiated CTRL and SDC-1 KD cultures, with GAPDH

as the loading control. Signal intensity was quantified using ImageJ software (NIH), error bar = SD. **(C)** Gene expression changes in adipogenic markers adiponectin (ADIPO-Q), CCAAT/enhancer binding protein alpha (C/EBPα), CCAAT/enhancer binding protein delta (C/EBPδ) and peroxisome proliferator-activated receptor gamma 1 (PPARγ1). Significant changes in gene expression was detected by Student's T test, significance level was set at *p<0.05. Error bars = SEM.

YU *et al.* - Supplementary Figure S4

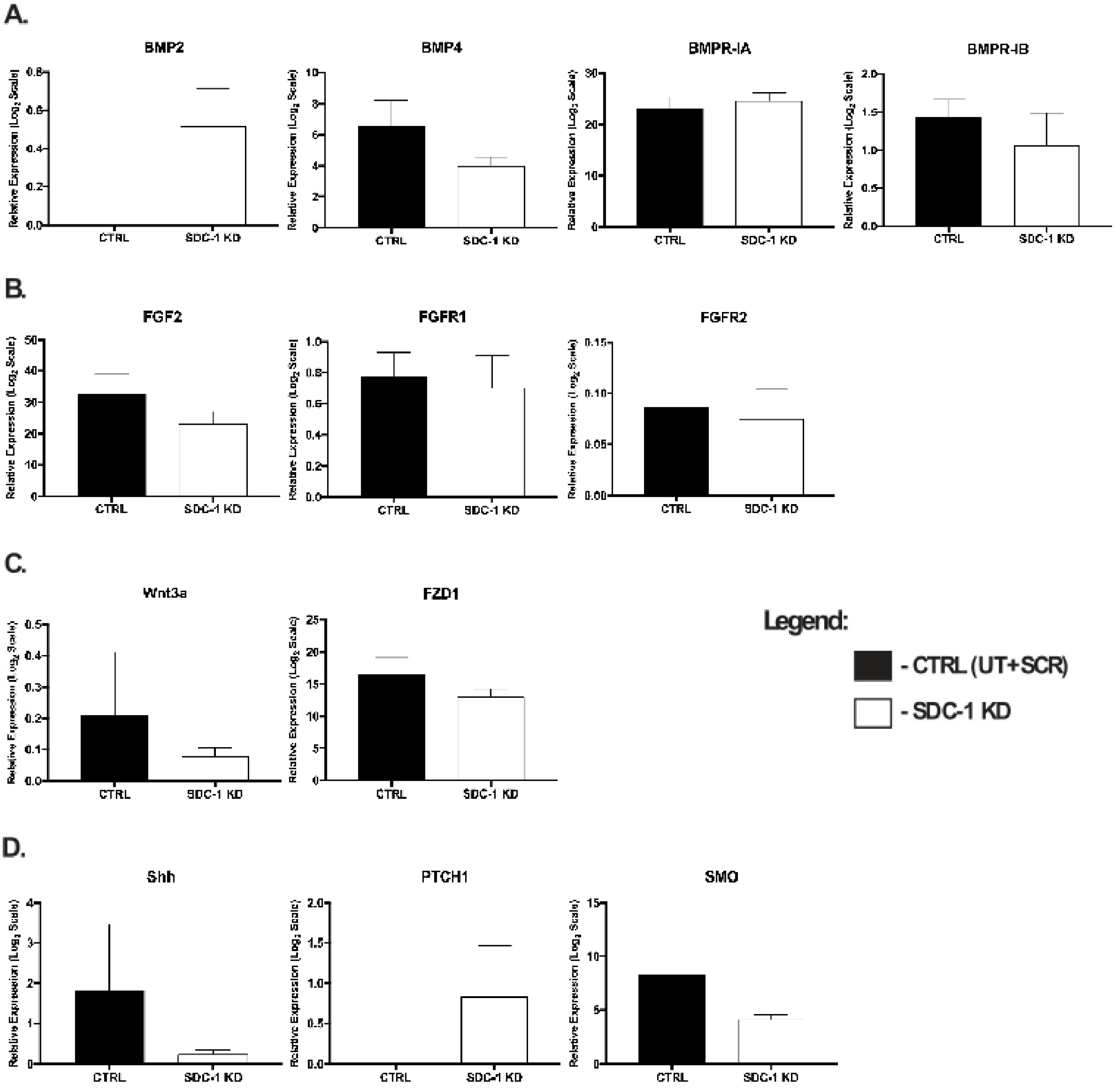

Supplementary Figure S4: Gene expression changes in common signalling pathways implicated in osteogenic and adipogenic lineages. Gene expression analysis was performed with RNA collected from hMSC incubated with SDC-1 specific siRNA for 72 h. Changes in gene expression in undifferentiated hMSC CTRL and SDC-1 KD cultures

detected by Q-PCR of **(A)** Bone morphogenetic protein (BMP) signalling including the ligands BMP2 and BMP4, and BMP receptors IA (BMPR-IA) and IB (BMPR-IB). **(B)** Fibroblast growth factor (FGF) signalling with ligand FGF2 and the receptors FGFR1 and FGFR2. **(C)** Wnt signalling including Wnt3a ligand and the receptor frizzled (FZD1). **(D)** Sonic hedgehog signalling (SHH) including the ligand SHH, the canonical receptor Patched (PTCH1) and the G protein-coupled receptor-like receptor Smoothened (SMO). Error bars represent SEM.

YU *et al.* - Supplementary Figure S5

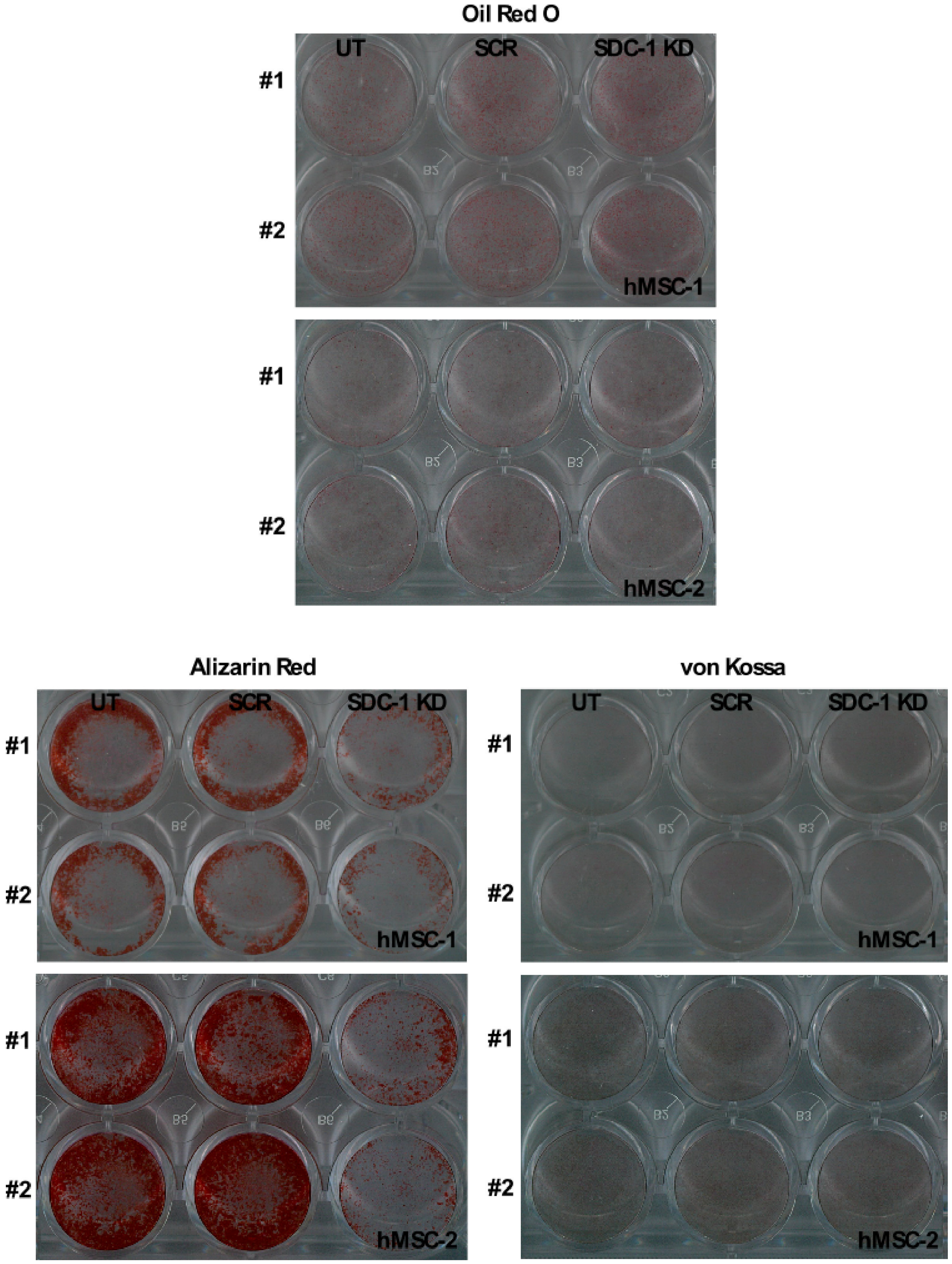

Supplementary Figure S5: Replicates of hMSC P+7 SDC-1 $KD_{AD/OS}$ osteogenic and adipogenic stained cultures. Terminal differentiated (22 days) hMSC SDC-1 KD_{AD} adipogenic cultures stained with Oil Red O, and the terminal differentiated (21 days) hMSC SDC-1 KD_{OS} osteogenic cultures stained with Alizarin Red and von Kossa. Two hMSC populations were used for SDC-1 KD differentiation experiments (hMSC-1 and hMSC-2). Differentiation of each hMSC population was performed in duplicate (#1 and #2) for each condition: Untreated ($UT_{AD/OS}$), Scrambled ($SCR_{AD/OS}$) and SDC-1 knockdown (SDC-1 $KD_{AD/OS}$).

YU *et al.* - Supplementary Figure S6

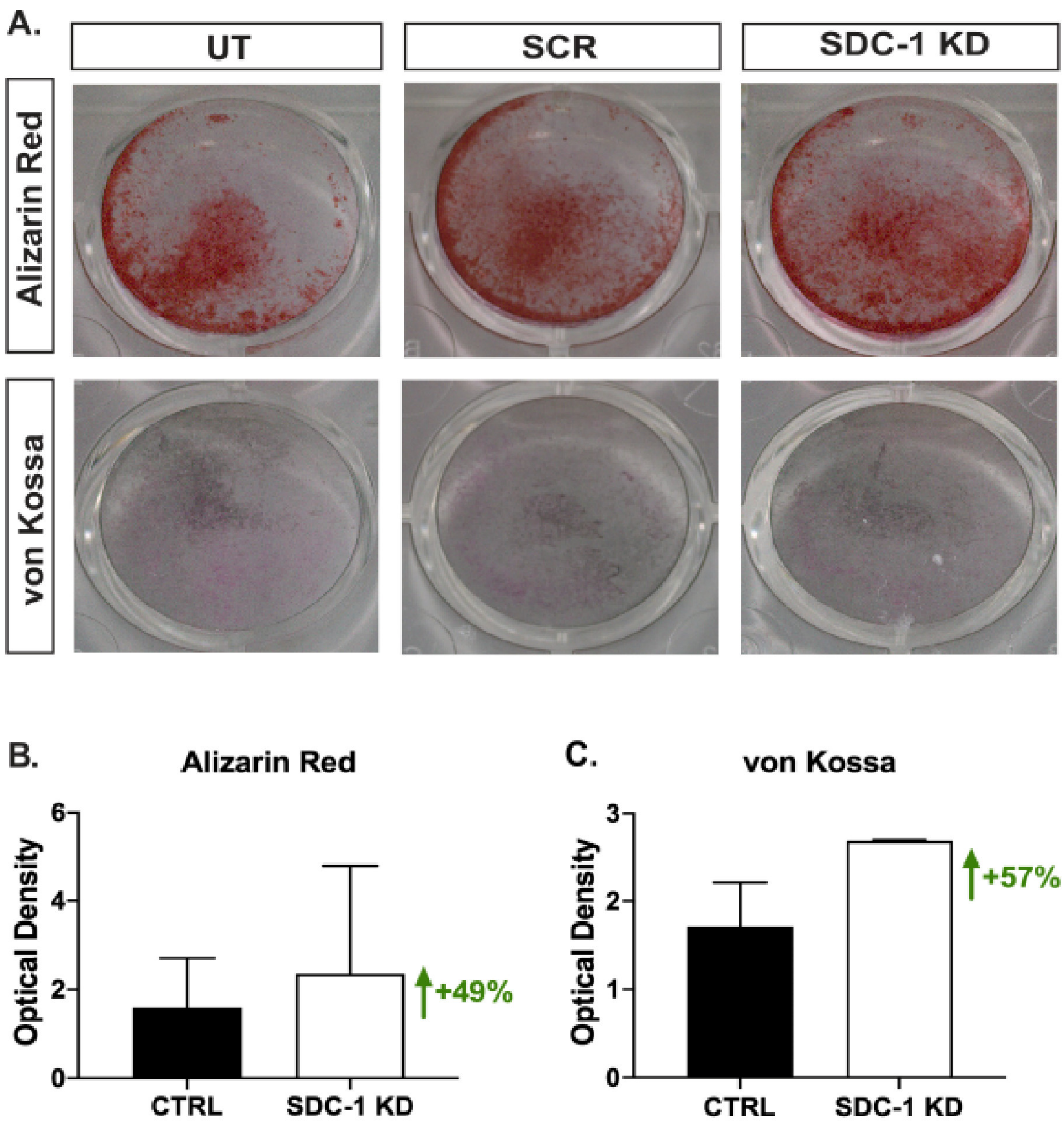

Supplementary Figure S6: Comparison of calcification and mineralisation between Untreated (UT), Scrambled (SCR) and SDC-1 knockdown (SDC-1 KD) hMSC post-KD osteogenic differentiated cultures by von Kossa and Alizarin Red staining, respectively. SDC-1 KD was performed in basal hMSC cultures, then these cultures were differentiated towards the osteogenic lineage, where terminally differentiated cultures (21 days) were examined by staining. Staining of hMSC SDC-1 KD osteogenic cultures by **(A)** Alizarin Red (red) and von Kossa stain (brown-black) with nuclear fast red-aluminium sulfate counter-stain (pink). Quantitation of **(B)** Alizarin Red (+49%) staining, and **(C)** von Kossa (+57%) in hMSC osteogenic SDC-1 KD cultures. Stained culture images were converted to 8-bit greyscale images and quantitated using ImageJ software (NIH). Optical density of CTRL cultures (UT and SCR averaged) and SDC-1 KD cultures was normalised to culture cell number recorded following SDC-1 KD, CTRL = 7.0×10^4 cells and SDC-1 KD = 5.0×10^4 cells (Fig. 1A).
